# Prevention of Conception

**Published:** 1915-08

**Authors:** 


**A Monthly Review of Medicine and Surgery**

EDITOR AND PUBLISHER, DR. A. L. BENEDICT, 228 Summer St., corner of Elmwood Ave., Buffalo, (Address for all communications. Please make personal and telephone calls before 1 P. M.)

**Third, in age, on the western continent. Independent, but supports professional organization. News limited mainly to Western N. Y. and Penn.**

**Subscription $2.00 a year; $3.00 for two years in advance. Changes and errors in address or failure or duplication of delivery, should be reported immediately.**

Yearly Volume 71. AUGUST, 1915. No. 1

### Prevention of Conception

The Critic & Guide reproaches medical journals with timidity in discussing this issue. The Medical Summary explains that the "outside of the pen looks good to most of us." It is a matter of common knowledge, lay as well as medical, that prevention is quite generally practiced. Vital statistics alone show that it is, on the whole, efficiently and safely practiced. All physicians occasionally note instances in which it is practiced both inefficiently and dangerously, and know that its inefficient use is often followed by more or less efficient and more or less dangerous attempts at abortion. Sociologists are taking up the matter from the economic side and many favor it not only as a matter of direct economics but as a eugenic factor in the sense that fewer children are better reared. There are cases in which the prevention of conception is professionally recognized as almost or quite life saving—excluding the obvious and often impractical alternative. There are many more cases in which its temporary use is professionally regarded as favorable to the economics and health of the family and thus, ultimately, of the community. We feel that the time is ripe for an abandonment of the policy of sticking our heads in the sand to blind ourselves to facts. Even the ostrich does not do this. He gets a good look and then braces himself to kick. We believe that when a method, however objectionable ethically, is in common use, it should be freed, so far as possible from danger and that, if used at all, it should be used intelligently. The law should recognize actual conditions, rather than theories. We speak thus, not from immorality but from a realization of the terrible catastrophies that attend blind ignorance and legendary, half understood, dilutions of fact. But, all sides of the question should be thoroughly discussed before propaganda for repeal of existing laws are in order; they should be discussed much more thoroughly still before propaganda for a professional and lay campaign are instituted. The medical practitioner and the medical editor must await the verdict of the legislator, the legislator must express the well-digested opinion of the economist and sociologist. The latter must consider well the opinion of those qualified to voice ethical, moral and religious opinion.

